# End-expiratory lung volume during mechanical ventilation: a comparison with reference values and the effect of positive end-expiratory pressure in intensive care unit patients with different lung conditions

**DOI:** 10.1186/cc7125

**Published:** 2008-11-20

**Authors:** Ido G Bikker, Jasper van Bommel, Dinis Reis Miranda, Jan Bakker, Diederik Gommers

**Affiliations:** 1 Department of Intensive Care Medicine, Erasmus MC, 's Gravendijkwal 230, 3015 CERotterdam, The Netherlands

## Abstract

**Introduction:**

Functional residual capacity (FRC) reference values are obtained from spontaneous breathing patients, and are measured in the sitting or standing position. During mechanical ventilation FRC is determined by the level of positive end-expiratory pressure (PEEP), and it is therefore better to speak of end-expiratory lung volume. Application of higher levels of PEEP leads to increased end-expiratory lung volume as a result of recruitment or further distention of already ventilated alveoli. The aim of this study was to measure end-expiratory lung volume in mechanically ventilated intensive care unit (ICU) patients with different types of lung pathology at different PEEP levels, and to compare them with predicted sitting FRC values, arterial oxygenation, and compliance values.

**Methods:**

End-expiratory lung volume measurements were performed at PEEP levels reduced sequentially (15, 10 and then 5 cmH_2_O) in 45 mechanically ventilated patients divided into three groups according to pulmonary condition: normal lungs (group N), primary lung disorder (group P), and secondary lung disorder (group S).

**Results:**

In all three groups, end-expiratory lung volume decreased significantly (*P *< 0.001) while PEEP decreased from 15 to 5 cmH_2_O, whereas the ratio of arterial oxygen tension to inspired oxygen fraction did not change. At 5 cmH_2_O PEEP, end-expiratory lung volume was 31, 20, and 17 ml/kg predicted body weight in groups N, P, and S, respectively. These measured values were only 66%, 42%, and 34% of the predicted sitting FRC. A correlation between change in end-expiratory lung volume and change in dynamic compliance was found in group S (*P *< 0.001; *R*^2 ^= 0.52), but not in the other groups.

**Conclusions:**

End-expiratory lung volume measured at 5 cmH_2_O PEEP was markedly lower than predicted sitting FRC values in all groups. Only in patients with secondary lung disorders were PEEP-induced changes in end-expiratory lung volume the result of derecruitment. In combination with compliance, end-expiratory lung volume can provide additional information to optimize the ventilator settings.

## Introduction

Monitoring end-expiratory lung volume (EELV) might be a valuable tool to optimize respiratory settings in mechanical ventilation [1]. However, determining EELV at the bedside in critically ill patients is not without difficulties. EELV can be measured using computed tomography [2,3], but this technique is not available for routine application at the bedside. Traditionally, EELV measurement techniques are based on dilution of tracer gases, such as sulfur hexafluoride washout [4], closed circuit helium dilution [5], or open circuit multi-breath nitrogen washout [6]. All of these techniques still need expensive and/or complex instrumentation and are in general not suitable for routine EELV measurements in the ICU. An alternative is the simplified helium dilution method, using a re-breathing bag with a helium mixture. However, an important disadvantage of this technique is that it requires interruption of mechanical ventilation for a short period of time [7]. Recently, Stenqvist and colleagues [8] introduced a novel method to measure EELV without interruption of mechanical ventilation, based on a simplified and modified nitrogen multiple breath washout (NMBW) technique, which is integrated into a mechanical ventilator. This method requires a step change in the inspired oxygen fraction (Fio_2_), without the need for supplementary tracer gases or specialized additional monitoring equipment [8].

Functional residual capacity (FRC) during spontaneous breathing is normally measured in the sitting or standing position and is length and age dependent. It has been shown that FRC is decreased by 25% in spontaneous breathing healthy volunteers after changing from the sitting to the supine position [9].

In critically ill patients receiving mechanical ventilation, FRC is determined by the level of positive end-expiratory pressure (PEEP), and it is therefore better to speak of EELV. Application of higher levels of PEEP leads to increased EELV values as a result of recruitment or further distention of already ventilated alveoli. To differentiate between recruitment and distention, EELV changes are combined with compliance values.

In this study, we used the modified NMBW technique to measure EELV at three different PEEP levels in mechanically ventilated patients with either non-acute respiratory failure or with a primary or secondary lung disorder. The results were compared with reference predicted FRC values, arterial oxygenation, and dynamic compliance.

## Materials and methods

The study population consisted of a convenient sample of 45 sedated and mechanically ventilated patients. For all patients, chest radiographs and, if available, computed tomography scans were retrospectively evaluated and related to clinical history and data to divide the patients into three groups: patients without acute respiratory failure (group N), those with respiratory failure due to primary lung disorders (group P), and those with respiratory failure due to secondary lung disorders (group S). With the approval of the local institutional human investigations committee, and after obtaining written informed consent, patients were enrolled in this study within 48 hours after intubation. Exclusion criteria were as follows: pneumothorax, pneumectomy, lung transplantation, and severe cardiovascular instability. Also, severe airflow obstruction due to chronic obstructive pulmonary disease (defined as forced expired volume in 1 second or vital capacity below predicted value minus 2 standard deviations) and patients with major inhomogeneous alveolar ventilation, as indicated by a significant upslope in phase III of the capnogram, were excluded. This was because gas wash out/in time could possibly be too short and end-tidal carbon dioxide could become unstable, potentially leading to errors in EELV measurement. We were unable to include patients with severe acute respiratory distress syndrome requiring a PEEP of 20 cm H_2_O in our protocol because a pressure limitation of the COVX module (GE Healthcare, Helsinki, Finland) at around 18 to 20 cmH_2_O PEEP.

During the study period patients were ventilated with an Engström Carestation ventilator (GE Healthcare, Madison, USA). EELV measurements were carried out with the COVX module (GE Healthcare, Helsinki, Finland) integrated within the ventilator. This module was described in detail previously [9]. At baseline, patients were switched to the Engström ventilator and ventilated according to their original settings before any measurements were performed. PEEP was increased to 15 cm H_2_O and the inspiratory pressure was adjusted to maintain tidal volume and without changing other ventilator settings. After a steady state had been achieved for at least 20 minutes, EELV was measured twice (wash-out and wash-in). This was repeated after a steady state lasting 10 minutes at both PEEP 10 cmH_2_O and PEEP 5 cmH_2_O. In all patients the same sequence of PEEP steps was used. Before each EELV measurement, hemodynamic and ventilatory parameters were recorded and arterial blood gas analysis performed (ABL 700; Radiometer, Copenhagen, Denmark) in order to calculate the arterial oxygen tension (Pao_2_)/Fio_2 _ratio. Arterial blood samples were taken 10 minutes after the PEEP change and just before the EELV measurement to avoid any influence of the step change in Fio_2 _required for the nitrogen wash-out/wash-in test. At the time of the EELV measurement, no muscle relaxation was used in the patients evaluated.

In all patients, EELV values were indexed according to predicted body weight (PBW) using the ARDSnet formula [10], which was calculated for men as 50 + 0.91 × (height [cm] – 152.4), and for women as 45.5 + 0.91 × (height [cm] – 152.4). In order to compare EELV values, reference EELV was calculated for each patient. Predicted sitting EELV was calculated for men as 2.34 × height (m) + 0.009 × age (years) – 1.09, and for women as 2.24 × height (m) + 0.001 × age (years) – 1.00 [11].

### Statistical analysis

Statistical analysis was performed with SPSS version 14.0 (SPSS Inc., Chicago, IL, USA). Data are expressed as mean ± standard deviation. Comparisons between the three groups were performed using one-way analysis of variance. When appropriate, *post hoc *analyses were performed with Bonferroni's test. To test whether and how EELV and Pao_2_/Fio_2 _ratio decreased with lower PEEP levels, we used analysis of variance for repeated measurements. Again, Bonferroni's test was used for *post hoc *analyses if appropriate. Correlation between EELV and the Pao_2_/Fio_2 _ratio or dynamic compliance was analyzed using Pearson's correlation. For all comparisons *P *< 0.05 was considered significant.

## Results

We examined 45 mechanically ventilated patients, retrospectively divided into three groups. Group N (n = 19) consisted of patients with traumatic brain injury (seven), cerebrovascular accident (seven), postoperative condition after neurosurgery (three), Fournier gangrene without evidence for pulmonary complications (one), and diagnostic laparotomy, without evidence for intra-abdominal hypertension (one). Group P (n = 16) consisted of patients with pneumonia (12), aspiration pneumonia (three) and major atelectasis (one). In group S (n = 10) all patients had abdominal sepsis. In the latter group, three out of the 10 patients had an open abdomen after decompression for intra-abdominal hypertension; the remainder of the patients with abdominal sepsis had an intra-abdominal pressure ranging from 10 to 15 cmH_2_O. Patient's baseline data were comparable between the three groups, except for Lung Injury Score and baseline PEEP. Baseline Pao_2_/Fio_2 _ratio and baseline dynamic compliance were lower in the two groups with lung disorders (groups P and S; Table 1).

**Table 1:** Data on the study patients by subgroup

Parameter	Normal lung function (group N)	Primary lung disorder (group P)	Secondary lung disorder (group S)
*n*	19	16	10

Female sex (%)	36.8	31.3	30.0

Age (years)	49 ± 16	52 ± 17	52 ± 18

Height (cm)	176 ± 9	177 ± 10	169 ± 6

Weight (kg)	73.6 ± 11.6	76.4 ± 15.0	78.7 ± 27.3

PBW (kg)	69.8 ± 10.6	71.0 ± 10.8	64.4 ± 7.2

LIS	0.9 ± 0.5	2.4 ± 0.8	2.1 ± 0.3

Tint (hours)	20.2 ± 16.6	28.9 ± 46.9	30.1 ± 26.2

Survival (*n*/*n *[%])	16/19 (84%)	13/16 (81%)	4/10 (40%)

Baseline PEEP (cmH_2_O)	6.2 ± 2.1	11.3 ± 4.1**	11.1 ± 2.6**

Baseline Pao_2_/Fio_2 _ratio (kPa)	49.7 ± 11.9	26.1 ± 11.2**	32.7 ± 13.1*

Baseline compliance dynamic (ml/cmH_2_O)	50.3 ± 13.0	35.6 ± 12.1*	38.8 ± 12.2*

Predicted sitting EELV (l)	3.3 ± 0.4	3.4 ± 0.4	3.2 ± 0.2

Ventilation mode			

Pressure control	7	8	4

Pressure support	5	6	6

Volume control	1	0	0

Pressure controlled – volume guaranteed	6	2	0

Unless otherwise stated, values are presented as mean ± standard deviation. The LIS (Murray) is based on dynamic compliance. *P = 0.05, **P = 0.001, versus group N. LIS, Lung Injury Score; PBW, predicted body weight; PEEP, positive end-expiratory pressure; Tint, time between intubation and inclusion.

**Figure 1:**
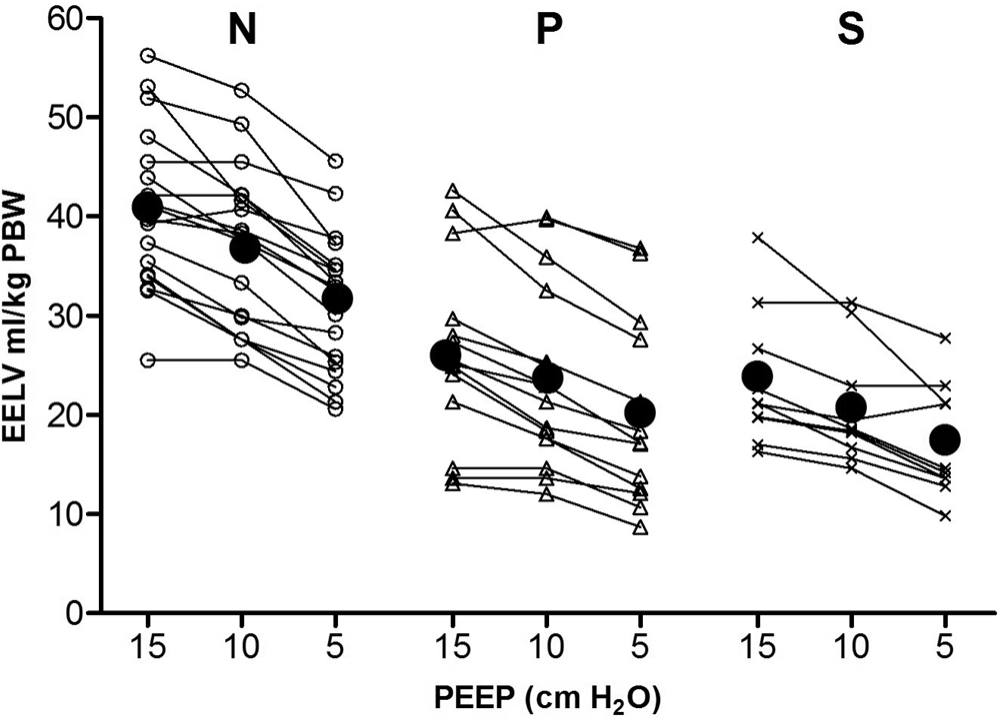
**Progression of EELV in individual patients over three stepwise reductions in PEEP**. Mean EELV values at each PEEP level are presented as black dots. Patients are divided according to the type of lung condition. Patients in group N had normal lungs, those in group P had a primary lung disorder, and those in group S had a secondary lung disorder. EELV, end-expiratory lung volume; PBW, predicted body weight; PEEP, positive end-expiratory pressure.

**Figure 2:**
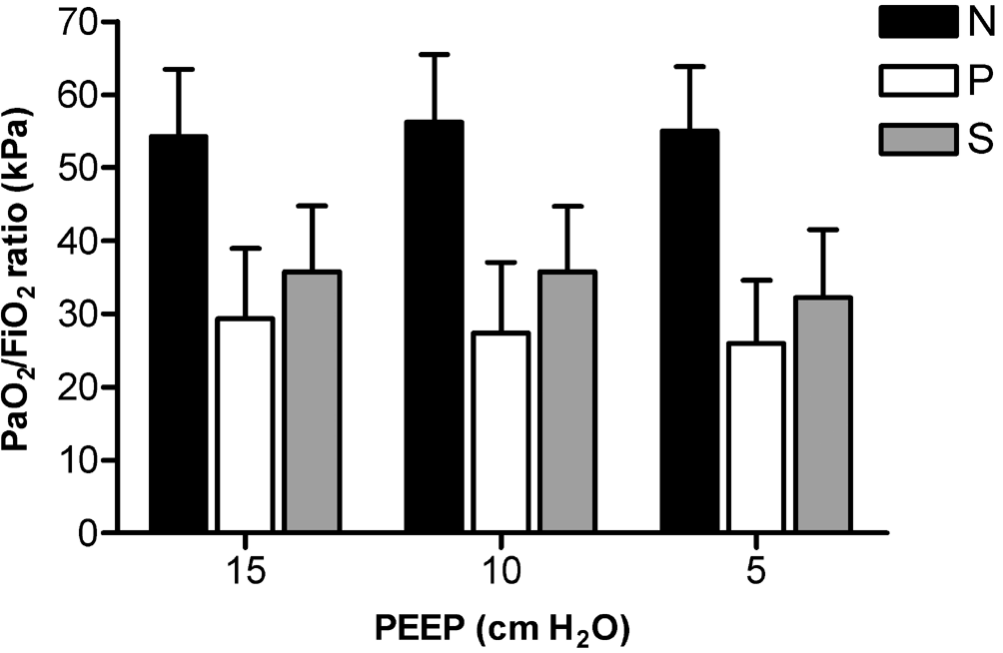
**Measured EELV as percentage of predicted sitting FRC at three PEEP levels**. The black dotted line represent predicted sitting FRC (100%). Patients in group N had normal lungs, those in group P had a primary lung disorder, and those in group S had a secondary lung disorder. Values are expressed as mean ± standard deviation. EELV, end-expiratory lung volume; FiO_2_, inspired oxygen fraction; FRC, functional residual capacity; Pao_2_, arterial oxygen tension; PEEP, positive end-expiratory pressure.

Measured EELV is presented in Figure 1. In group N, measured EELV at 5 cmH_2_O PEEP was 66% of the predicted sitting FRC (Figure 2). In both groups with lung disorders (groups P and S), EELV was significantly (*P *< 0.001) reduced to 42%, and 35% of the predicted sitting FRC, respectively. Mean EELV values at 15, 10, and 5 cmH_2_O PEEP were 40.9, 37.1, and 31.3 ml/kg PBW, respectively, in group N; 26.0, 23.6, and 20.2 ml/kg PBW in group P; and 23.4, 20.6, and 17.2 ml/kg PBW in group S.

**Figure 3:**
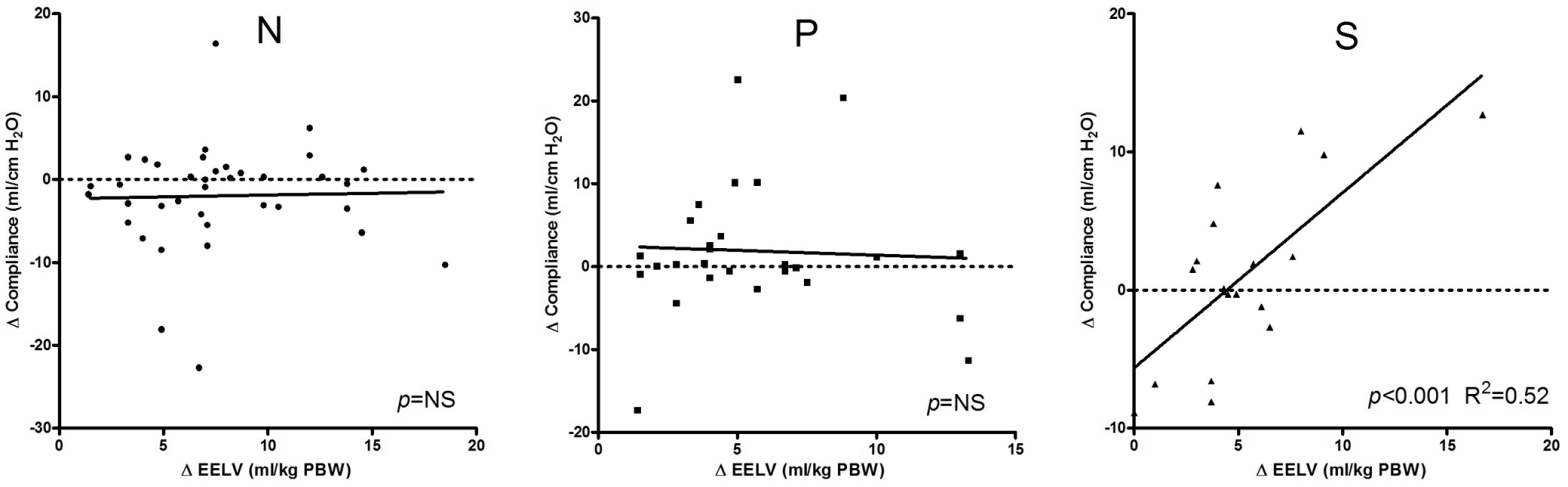
**Pao_2_/Fio_2 _ratio in different types of lung conditions at three PEEP levels**. Patients in group N had normal lungs, those in group P had a primary lung disorder, and those in group S had a secondary lung disorder. Values are expressed as mean ± standard deviation. EELV, end-expiratory lung volume; FiO_2_, inspired oxygen fraction; Pao_2_, arterial oxygen tension; PBW, predicted body weight; PEEP, positive end-expiratory pressure.

**Figure 4:**
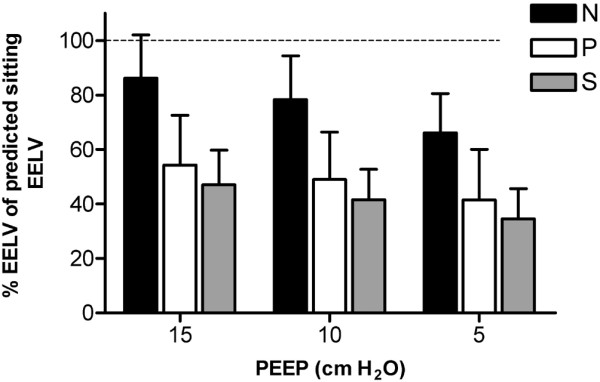
**Correlation between change in EELV and change in dynamic compliance**. Data are presented as the difference between the lowest PEEP level (5 cmH_2_O) and 10 or 15 cmH_2_O PEEP. Patients in group N had normal lungs, those in group P had a primary lung disorder, and those in group S had a secondary lung disorder. EELV, end-expiratory lung volume; PEEP, positive end-expiratory pressure.

The effect of the stepwise reduction in PEEP on the change in EELV in each patient in the three study groups is shown in Figure 1. Irrespective of group, EELV decreased linearly with reductions in PEEP; only in some patients was an increase or decrease in the slope observed after stepwise reduction in PEEP level. In all three groups, EELV decreased significantly (*P *< 0.001) while decreasing PEEP from 15 to 5 cm H_2_O, whereas the Pao_2_/Fio_2 _ratio did not change (Figures 1 and 3). Patients in group S had lower EELV, but higher Pao_2_/Fio_2 _ratio, compared with group P (Figures 1 and 3). EELV was correlated with the Pao_2_/Fio_2 _ratio in group P (*R*^2 ^= 0.40; *P *= 0.02), but not in groups N and S. Correlation between change in EELV and change in compliance was significant in group S (*P *< 0.001; *R*^2 ^= 0.52), but not in groups N (*P *= 0.51) and P (*P *= 0.94; Figure 4).

## Discussion

In mechanically ventilated patients with and without acute respiratory failure, measured EELV was markedly reduced in comparison with the predicted sitting FRC. Only in patients with secondary lung disorders were EELV changes accompanied by compliance changes, indicating derecruitment after reducing the PEEP. In addition, we did not identify a good correlation between measured EELV and the Pao_2_/Fio_2 _ratio in any of the three study groups.

Blood gases are frequently used to monitor the patient's lung function during mechanical ventilation. One should note that determining lung collapse by Pao_2_/Fio_2 _ratio assumes minimal extrapulmonary shunt. Cressoni and coworkers [12] have shown that variation in gas exchange cannot be used with sufficient confidence to assess anatomical lung recruitment in patients with acute lung injury (ALI)/acute respiratory distress syndrome (ARDS). It therefore seems reasonable to monitor lung volume changes caused by alveolar recruitment or alveolar collapse by repeated measurements of FRC instead of arterial oxygenation. FRC is defined as the relaxed equilibrium volume of the lungs when there is no muscle activity and no pressure difference between alveoli and the atmosphere [13]. FRC is determined in spontaneously breathing, resting normal individuals at the end of a normal expiration, and therefore EELV is used to denote 'FRC' during mechanical ventilation.

Most studies addressing EELV in the ICU describe new techniques with good accuracy and good repeatability, but without presenting their data on the measured EELV values for the individual ICU patient [5-7,14,15]. Olegard and colleagues [8] measured EELV in a mixed ICU population and found EELV volumes ranging from 1,153 to 5,468 ml, but they did not report on the PEEP levels used. Only Neumann and coworkers [16] presented the measured mean EELV data for postoperative patients, and patients with ALI and chronic obstructive pulmonary disease at different PEEP levels (0, 5, and 10 cmH_2_O). In their study, at a PEEP of 5 cmH_2_O the measured EELV values were 2.5 l and 1.5 l in the postoperative and ALI groups, respectively. We found comparable EELV data for the similarly defined groups of patients (groups N and P) at comparable PEEP levels.

Normally, FRC reference values are obtained from spontaneously breathing patients in the standing or sitting position [11], but no reference values are available for supine mechanically ventilated patients. Ibanez and colleagues [9] showed that FRC decreased by 25% after changing the patient's position from sitting to supine during spontaneous breathing in healthy volunteers. If one assumes that ventilation of a 'healthy' lung at a PEEP of 5 cmH_2_O occurs approximately at FRC level, then we found a reduction of 34% in group N (measured EELV compared with predicted sitting FRC). This extra reduction in EELV (34% versus 25%) is probably due to loss of muscle tension attributed to the use of sedation in our ICU patients. Furthermore, we opted not to use the regression equations of Ibanez and colleagues [9] to calculate predicted supine FRC for our patients, because their study population consisted of relatively short (mean 1.65 m) and young people (mean 35 years), and age was not included in their regression equations, whereas our ICU population consisted mainly of tall, elderly people. Instead, we decided to use the predicted sitting FRCs [11] and to reduce these based on the reduction observed in patients without lung disorders at 5 cmH_2_O PEEP (34%) to estimate the predicted supine FRC. In groups P and S, measured EELV values were 63% and 53%, respectively, of the predicted supine FRC at a PEEP of 5 cmH_2_O.

EELV measurements alone cannot be used to define optimal ventilator settings, because EELV can be increased without recruitment (already open alveoli are further inflated). Therefore, increases in both EELV and dynamic compliance should be used to identify successful recruitment. In our study, we did not perform a recruitment maneuver but applied 15 cmH_2_O PEEP in all patients. In group N (without lung disorders), the Pao_2_/Fio_2 _ratio at 5 cmH_2_O PEEP was already 49.7 kPa (373 Torr), indicating that the lung was almost entirely open at this PEEP level and therefore application of higher PEEP levels would only further inflate the already open alveoli. Gatinnoni and coworkers [17] showed that ARDS from extrapulmonary origin had an abnormally increased chest wall elastance and a major response to the application of 15 cmH_2_O PEEP, whereas ARDS from primary pulmonary origin showed a lack of recruitment and an increase in total respiratory elastance with the application of PEEP. The group with primary lung disorders could be compared to ARDS from pulmonary origin with consolidation, whereas group S could be compared to ARDS from extrapulmonary origin with prevalent edema and lung collapse. In our study we found a significant correlation between EELV and compliance in group S, but not in groups N and P (Figure 4). This change in lung volume accompanied by compliance indicates recruitment or derecruitment. In this study, patients with secondary lung disorders benefitted from higher PEEP, whereas patients with primary or without lung disorders did not, and application of higher PEEP in this setting would lead to overdistention.

Surprisingly, patients with secondary lung disorders due to abdominal sepsis had the lowest EELV values at the PEEP levels we used (Figure 1). From obese patients, we have learned that increased intra-abdominal pressure leads to decreased chest wall compliance and a cranial shift of the diaphragm, with consequent reduction in lung volume and atelectasis formation, especially in the basal parts of the lung. In group P (patients with pneumonia), EELV was also decreased but this was due to consolidation in a part of the lung.

For our measurements we used the NMBW method with a step change of 0.2 in Fio_2 _to measure EELV. With this method, the alveolar EELV is calculated without the anatomical dead space [8]. We were able to perform stable measurements in both controlled and partial support ventilatory modes, and we found no significant difference in EELV between the two modes. Using this NMBW method, it is assumed that there is no transfer of nitrogen from alveoli to blood during the EELV measurement, but this can be eliminated by an EELV measurement during wash-out and one during wash-in.

## Conclusion

We conclude that in mechanically ventilated and sedated patients, EELV is markedly reduced compared with predicted sitting FRC values. In addition, it has become clear that PEEP-induced changes in EELV not only represent recruitment or derecruitment, but they can also be the result of inflation or deflation of already ventilated lungs. Therefore, EELV alone is not the 'magic' bullet, but in combination with compliance it can provide additional information to optimize the ventilator settings.

## Key messages

• EELV is markedly reduced in critically ill patients.

• EELV in ICU patients without lung disorders ventilated at 5 cmH_2_O PEEP is reduced with 34% compared with FRC reference values in sitting position.

• Compliance and EELV are correlated only in patients with respiratory failure because of secondary lung disorders, indicating successful recruitment.

• During mechanical ventilation, EELV in combination with compliance can provide additional information that can help in optimizing ventilator settings.

## Abbreviations

ALI: acute lung injury; ARDS: acute respiratory distress syndrome; EELV: end-expiratory lung volume; FiO_2_: inspired oxygen fraction; FRC: Functional residual capacity; NMBW: nitrogen multiple breath washout; Pao_2_: arterial oxygen tension; PEEP: positive end-expiratory pressure.

## Competing interests

We received an unrestricted grant from GE Healtcare.

## Authors' contributions

IB carried out the data acquisition, analysis, statistical analysis, and participated in drafting the manuscript. DRM participated in the statistical analysis and drafting the manuscript. DG participated in the data acquisition and drafting the manuscript. JvB and JB participated in drafting the manuscript. All authors read and approved the final manuscript.
